# Expression of Nuclear Receptor Coactivators in the Human Fetal Membranes at Term before and after Labor

**DOI:** 10.1155/2012/717294

**Published:** 2012-12-20

**Authors:** Che-Wei Ryan Ou, Meihua Sun, Weronika Sadej, William Gibb

**Affiliations:** ^1^Department of Obstetrics and Gynecology, University of Ottawa, The Ottawa Hospital, General Campus, 501 Smyth Road, Room 8420, Ottawa, ON, Canada K1H 8L6; ^2^Department of Cellular and Molecular Medicine, University of Ottawa, 451 Smyth Road, Ottawa, ON, Canada K1H 8M5

## Abstract

Human fetal membranes play an important role in term and preterm labor and are responsive to steroids. We examined the expression of steroid receptor coactivators in fetal membranes obtained prior to and following labor at term. Proteins were localized by immunohistochemistry, Western analysis was carried out in nuclear extracts, and mRNA levels were determined by real-time RT-PCR. SRC-1, SRC-2, p300, and PCAF proteins were present in all nuclear extracts. The amnion nuclei expressed higher levels of SRC-1, p300, and PCAF than nuclei from the chorion-decidua, whereas the reverse was true for SRC-2. Chorion-decidua from patients before labor expressed higher levels of SRC-1 than those from patients after labor. Also, the PCAF level was higher in the amnion obtained before labor than the same tissue obtained after labor. In contrast to the protein expression, mRNA levels of SRC-1 and p300 were higher in the chorion-decidua compared to the amnion, whereas there was no difference in levels of SRC-2 and PCAF mRNAs between these two tissues. These data underline that the regulation of the expression of the coactivators in these tissues occurs during labor and is complex and tissue specific.

## 1. Introduction

Preterm birth remains the leading cause of perinatal mortality and morbidity. Despite intensive efforts towards prevention, prompt diagnosis, and use of a variety of tocolytics, the incidence of preterm birth has increased [1]. This is in part due to lack of understanding of the mechanisms responsible for the initiation of labor. Understanding the molecular basis for labor is essential for the prevention, early diagnosis, and effective treatment of preterm labor.

The onset of labor in pregnant women involves multiple complex pathways including both endocrine and mechanical signals [2, 3]. In both the human and sheep, glucocorticoids and progesterone appear to play a central role in the parturition. In the fetal membranes, glucocorticoids increase the expression of type 2 prostaglandin H_2_ synthase (PGHS-2), a rate-limiting enzyme for the production of prostaglandins (PGs), in the amnion cells in culture [4, 5]. Glucocorticoids also inhibit the expression and activity of 15-hydroxyprostaglandin dehydrogenase (PGDH), the major PG degrading enzyme, in the human fetal membranes, and progesterone is believed to tonically stimulate expression of this enzyme [6]. Furthermore, glucocorticoids stimulate the expression of corticotropin-releasing hormone (CRH) in the fetal membranes and placenta [7, 8], and CRH has also been shown to stimulate PG production [9]. These processes may then result in a net increase in PG output from the fetal membranes, which plays a key role in cervical ripening and increased myometrial contractility prior to and during parturition [10]. Evidence suggests that all of these actions are regulated by the glucocorticoid receptor [11, 12]. 

Glucocorticoids regulate gene transcription by interacting with a specific nuclear receptor. The glucocorticoid receptor (GR), a member of the type I nuclear receptor family, is coupled to heat-shock proteins and sequestered to the cytoplasm in the absence of ligand [13]. Upon ligand binding, GR dissociates from the chaperones, dimerizes, and translocates to the nucleus where it recruits nuclear receptor coactivators, binds to steroid-response elements in the promoter region of the target genes, and affects gene transcription. Several classes of nuclear receptor coregulators have been identified and are essential in this steroid responsiveness [14, 15]. The p160 steroid receptor coactivator (SRC) family, consisting of SRC-1/NCoA-1, GRIP-1/TIF-2/SRC-2, and pCIP/RAC-3/ACTR/AIB-1/TRAM-1/SRC-3, functions by enhancing transcriptional activation and recruitment of other cofactors possessing potent histone acetyl transferase activity [16]. The latter class of coregulators includes p300/CBP and PCAF [17]. In addition, the chromatin-remodeling complexes such as SWI/SNF are also important in the transcriptional activity of GR. Brg1 and Brm are the ATPase subunits of this complex [18].

The expression and localization of GR in the human fetal membranes have been described previously. We have shown that GR protein was highly expressed in the nuclei of amnion epithelial cells, mesenchyme, and chorion laeve trophoblasts both at term and preterm and that GR was present, but less defined in the decidua [19]. However, no studies have examined the expression of steroid receptor coregulators in the human fetal membranes. Recent studies with the human term myometrium, however, have proposed an important role for steroid receptor coregulators in parturition in this tissue. Condon et al. demonstrated a decrease in the expression of the coactivators SRC-2 and SRC-3 and CBP expression in human fundal myometrium during labor and suggested that this may be responsible at least in part for functional progesterone withdrawal during labor in this tissue [20]. Also, studies by Dong et al. have suggested that changes in the expression of the progesterone receptor (PR) corepressor PSF may also be involved in the functional withdrawal of progesterone at parturition in the myometrium [21]. The objectives of the present study were to define the expression and localization of steroid receptor coactivators in the human fetal membranes at term and to determine whether changes in coactivator expression occurred in these tissues during labor, which may be indicative of potential changes in the action of steroids in these tissues at this time. 

## 2. Materials and Methods

### 2.1. Study Subjects and Tissue Collection

Twelve pregnant patients undergoing elective cesarean section without labor at term (≥37 weeks of gestation) and 12 patients having spontaneous vaginal delivery at term were recruited in the study and gave informed consent. The experimental protocol has been approved by the Ottawa Hospital Research Ethical Committee. Fetal membranes were removed 1 inch above the placental plate and the amnion separated from the chorion-decidua. These tissues were used for Western analysis and real-time RT-PCR. Tissues were frozen in dry ice-cooled isopentane and stored in −80°C for later analysis.

### 2.2. Isolation of Nuclear Extracts

Nuclear proteins were prepared from these tissues using a protocol that was described previously [22]. Tissues were minced and incubated in cold buffer L (10 mM Tris-HCl pH 7.4, 1.5 mM MgCl_2_, 10 mM KCl, 0.5 mM dithiothreitol (DTT), 0.5% NP-40, and Complete Protease Inhibitor Cocktail (Roche Diagnostics, Mannheim, Germany)) on ice for 10 min. Samples were homogenized using a Tissue Tearer (BioSpec Products, Inc.) at medium speed for 30 sec for 3 times with 30-sec coolings between bursts. The homogenates were then centrifuged at 1000 ×g for 10 min at 4°C. The pellets were resuspended in cold buffer N (50 mM Tris-HCl pH 7.4, 20% glycerol, 400 mM KCl, 1.5 mM MgCl_2_, 0.2 mM EDTA, 0.5 mM DTT, and Complete Protease Inhibitor Cocktail) and incubated on ice for 30 min. The supernatant was collected after centrifugation at 3000 × g twice for 5 min. Protein concentration was determined by the Bradford protein assay (BIO-RAD, Hercules, CA, USA).

### 2.3. Western Analysis

Eighty *μ*g of nuclear extracts were separated on 4–15% Tris-HCl SDS-PAGE (BIO-RAD). The proteins were transferred onto PVDF membranes (Millipore, Bedford, MA, USA) by running at 35 V at 4°C overnight. The membranes were blocked in 1 × phosphate-buffered saline-0.05% Tween-20 (PBS-T) containing 5% Carnation skim milk powder for 1 h. The membranes were then incubated with primary antibodies against SRC-1 (M-341), GRIP-1/SRC-2 (M-343), RAC3/SRC-3 (H-270), p300 (C-20), CBP (C-20), PCAF (H-369), Brg-1 (H-88), or Brm (N-19) diluted 1 : 500 in blocking solution at room temperature for 1 h. All of the above antibodies were from Santa Cruz Biotechnology, Inc. (Santa Cruz, CA, USA). Membranes were washed with 1 × PBS-T four times for 5 min and then incubated with secondary antibody diluted 1 : 2000 in blocking solution at room temperature for 1 h. Horseradish peroxidase-linked donkey anti-rabbit IgG (Amersham Biosciences, Buckinghamshire, UK) was used as a secondary antibody for all the reactions except for Brm, where donkey anti-goat IgG (Amersham Biosciences) was used. The filters were washed with 1 × PBS-T four times for 5 min, developed using Western Lightning Chemiluminescence Reagent Plus Kit (Perkin Elmer, Boston, MA), and exposed to Kodak Biomax MR films (Eastman Kodak, Rochester, NY, USA) for a sufficient time. Signals on the films were then scanned and subjected to densitometric analysis using Kodak Digital Science software. To normalize for loading differences, membranes were stained with Ponceau S, and a prominent 50-kD band was used as the internal loading control. No differences in levels of this protein were found between tissues obtained prior to and after labor. 

### 2.4. Real-Time RT-PCR

Real-time RT-PCR was carried out with the same methodology we used in previous studies [23, 24]. Total RNA was extracted from frozen tissues using TRIzol Reagent (Invitrogen Canada, Inc., Burlington, ON, Canada) according to the manufacturer's instructions and quantified spectrophotometrically. Total RNA was treated with TURBO DNase (Ambion, Austin, TX, USA) to remove residual DNA.

Reactions were performed by TaqMan One Step PCR Master Mix Reagent Kit and using ABI PRISM 7000 apparatus (Applied Biosystems, Foster City, CA, USA) according to the manufacturer's instructions. The primers and TaqMan probes were designed with Primer Express 2.0 software (Applied Biosystems, CA, USA). The sequences of these primers and probes for SRC-1, SRC-2, p300, and PCAF are shown in Table 1. RT-PCR reaction mixtures contained 1 × TaqMan Master Mix, 1 × MultiScribe and RNase inhibitor mix, 2 *μ*L (100 ng) cDNA template, 2.5 *μ*M of each primer, and 10 *μ*M of TaqMan probe. Reverse transcription reaction conditions were 48°C for 30 min and 95°C for 10 min. Polymerase chain reaction conditions were 40 cycles at 95°C for 15 sec and 60°C for 1 min. RT-PCR experiments were performed using serially diluted RNA to obtain a standard curve with 4 different concentrations in triplicate. From the standard curve, the copy number of each gene in the tissues was calculated. GAPDH, TaqMan GAPDH Control (Applied Biosystems), was chosen as an internal control. Data were normalized by using the ratio of the target cDNA concentration to that of GAPDH. For every run optimization of the primers for the coregulators and GAPDH was carried out using various dilutions of the probes in order to determine the appropriate dilutions for quantitative analysis. As in previous studies [24] the expression of GAPDH did not change during labor.

### 2.5. Immunohistochemistry

Unseparated amnion-chorion-decidual tissues, from an area of the membranes 1 inch above the placental plate and distant to the rupture site, were sectioned at 10 *μ*m on a cryostat at −22°C, thaw-mounted onto cooled Superfrost/Plus slides (Fisher Scientific, Nepean, ON, Canada), and stored at −70°C. The sections were postfixed by immersion in 4% paraformaldehyde for 5 min at room temperature and washed twice for 5 min in PBS, pH 7.4. The sections were then stained for SRC-1, SRC-2, p300, and PCAF using an Elite Vectastain ABC Kit (Vector Laboratories, Burlington, CA, USA). Sections were incubated in 0.3% H_2_O_2_ in PBS for 30 min to quench endogenous peroxidase activity and washed in PBS for 10 min. The sections were incubated with 10% normal serum in PBS for 30 min to block nonspecific binding, and excess serum was removed by blotting. Primary antibodies as described in Western analysis were diluted in PBS containing 2% normal serum. The dilutions were 1 : 1,000 for SRC-1, 1 : 200 for SRC-2, and 1 : 500 for p300 and PCAF antisera. This was applied to the section and incubated at 4°C for 18–20 h. Slides were then brought to room temperature, washed three times with PBS for 5 min, then incubated with biotinylated secondary antibody for 1 h, and washed three times with PBS for 5 min. Avidin-biotin-peroxidase complex in PBS was applied for 1 h. After washing in PBS three times for 5 min, the sections were incubated in diaminobenzidine tetrahydrochloride (DAB) peroxidase substrate solution (SIGMA FAST 3,3′-diaminobenzidine tablet sets) (Sigma Chemical Co., St Louis, MO) for 2–5 min. Slides were then dehydrated through graded series of ethanol, cleared in xylene substitute, and mounted with Permount (Fisher Scientific). No counterstain was applied. No staining was found in control slides incubated with nonimmune IgG.

### 2.6. Statistical Analysis

Data from Western analysis and real-time RT-PCR were expressed as the mean ± SEM. Data were subjected to two-way ANOVA. Comparisons between nonlabor and labor groups were performed in the amnion and in the chorion-decidua separately using Student's *t*-test. When data did not follow a normal distribution, Mann-Whitney rank sum test was performed. Statistical analysis was carried out using SigmaStat version 1.01 software (Jandel Corporation, San Rafael, CA, USA). The level of significance for comparison was set at  *P* < 0.05.

## 3. Results

**Figure 1:**
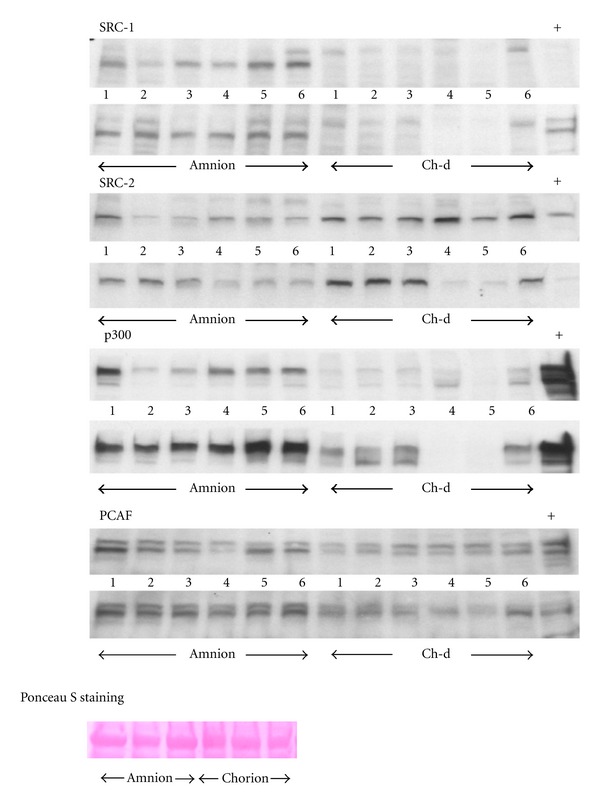
Representative Western blots of amnion and chorion-decidua (Ch-d) of six different tissues from non-labor patients undergoing elective cesarean section (C/S) and six labor patients after spontaneous vaginal delivery. Blots were probed with antisera specific to SRC-1, SRC-2, p300, and PCAF. Nuclear extracts from K562 cells (Santa Cruz) were used as a positive control for these proteins (+). Lanes marked 1–3 represent C/S tissues and 4–6 represent labor tissues. Typical Ponceau S staining, used as loading control, is shown in the lower panel.

Western analysis of nuclear proteins from human fetal membranes showed that SRC-1, SRC-2, p300, and PCAF were expressed in the nuclear extracts from the amnion and chorion-decidua (Figure 1). However, SRC-3, CBP, Brg-1, and Brm were not detectable.

### 3.1. SRC-1

**Figure 2:**
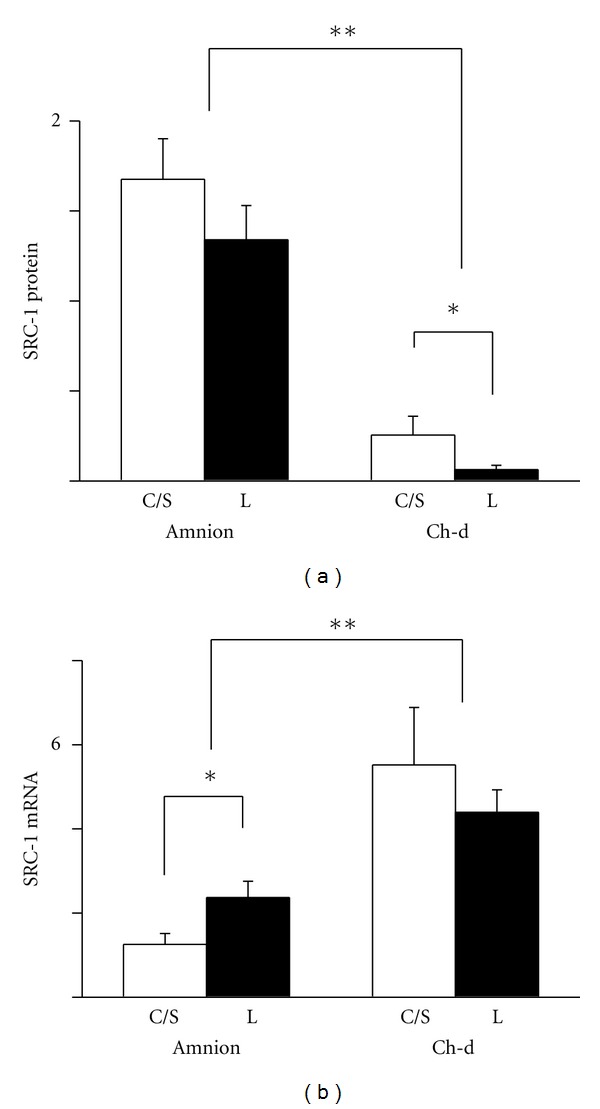
Expression of SRC-1 protein (a) and mRNA (b) in the amnion and chorion-decidua (Ch-d) from non-labor group (C/S) and labor group (L). Data were presented as the mean ± SEM. (a) **P < 0.05 between amnion and chorion-decidua. *P < 0.05 between C/S group and labor group within the chorion-decidua. (b) **P < 0.05 between amnion and chorion-decidua. *P < 0.05 between C/S group and labor group within the amnion. Ponceau S staining was used as the internal control for Westerns and GAPDH mRNA as the internal control for mRNA.

SRC-1 protein level was higher in the amnion nuclei compared to the chorion-decidua nuclei. There was no difference between non-labor and labor groups in the amnion, but in the chorion-decidua, the level was higher in the non-labor group when compared to the labor group (Figure 2(a)). We also investigated the mRNA levels of SRC-1 in these tissues by real-time RT-PCR. In contrast to the protein expression, SRC-1 message level was higher in the chorion-decidua than in the amnion. The non-labor group expressed lower SRC-1 message level compared to the labor group only in the amnion (Figure 2(b)). 

### 3.2. SRC-2

**Figure 3:**
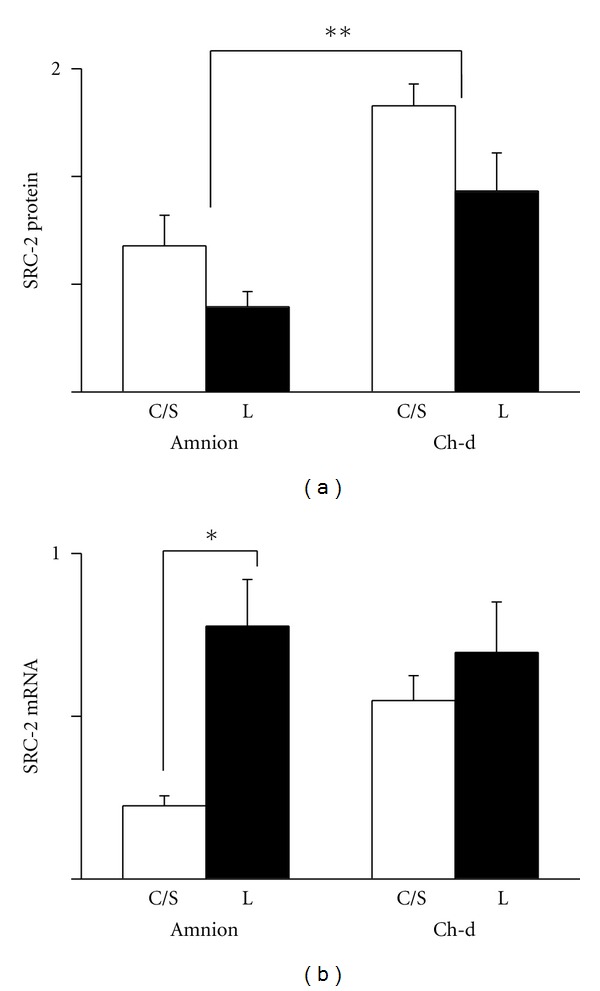
Expression of SRC-2 protein (a) and mRNA (b) in the amnion and chorion-decidua (Ch-d) from non-labor group (C/S) and labor group (L). Data were presented as the mean ± SEM. (a) **P < 0.05 between amnion and chorion-decidua. (b) *P < 0.05 between C/S group and labor group within the amnion. Ponceau S staining was used as the internal control for Westerns and GAPDH mRNA as the internal control for mRNA.

Analysis of protein levels of SRC-2 in the fetal membranes revealed a higher expression in the chorion-decidua than in the amnion. Comparisons between non-labor and labor groups within the amnion or chorion-decidua did not show a statistical difference (Figure 3(a)). Using real-time RT-PCR, the message levels of SRC-2 did not differ between the amnion and chorion-decidua. However, individual comparisons showed that in the amnion, the level was higher in the labor group than in the non-labor group (Figure 3(b)).

### 3.3. p300

**Figure 4:**
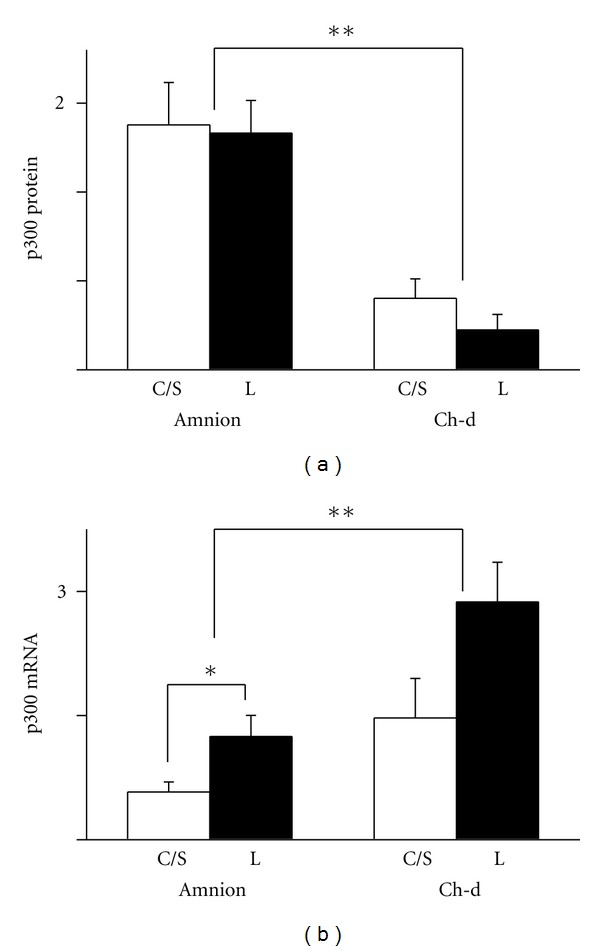
Expression of p300 protein (a) and mRNA (b) in the amnion and chorion-decidua (Ch-d) from non-labor group (C/S) and labor group (L). Data were presented as the mean ± SEM. (a) **P < 0.05 between amnion and chorion-decidua. (b) **P < 0.05 between amnion and chorion-decidua. *P < 0.05 between C/S group and labor group within the amnion. Ponceau S staining was used as the internal control for Westerns and GAPDH mRNA as the internal control for mRNA.

The amnion expressed higher levels of p300 protein than the chorion-decidua. Comparisons within the tissues failed to demonstrate a difference between the tissues obtained before and after labor (Figure 4(a)). In contrast, p300 mRNA level was higher in the chorion-decidua than in the amnion. However, the level was higher in the labor group compared to the non-labor group only in the amnion but not in the chorion-decidua (Figure 4(b)). 

### 3.4. PCAF

**Figure 5:**
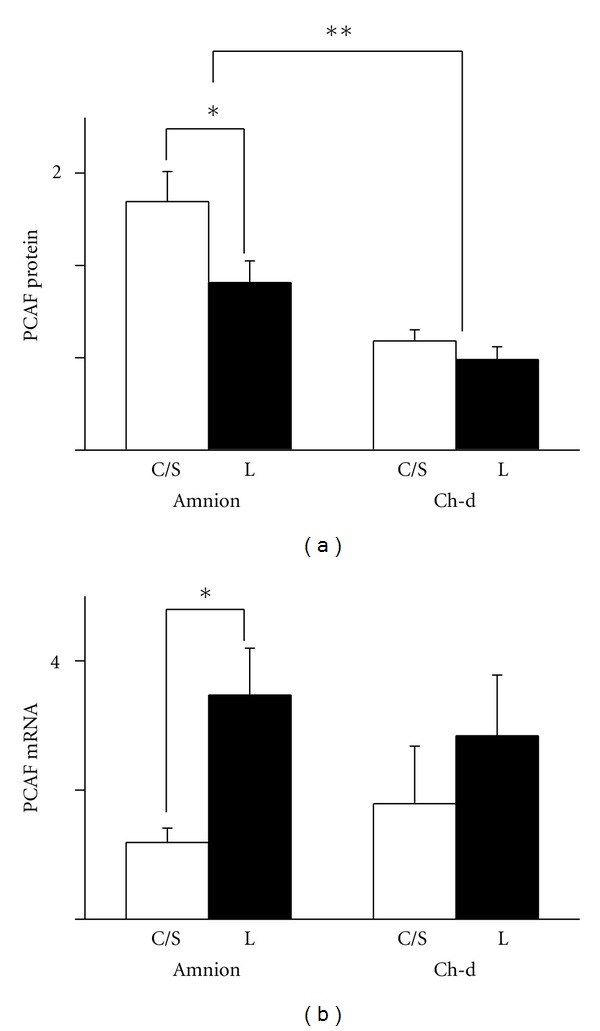
Expression of PCAF protein (a) and mRNA (b) in the amnion and chorion-decidua (Ch-d) from non-labor group (C/S) and labor group (L). Data were presented as the mean ± SEM. (a) **P < 0.05 between amnion and chorion-decidua. *P < 0.05 between C/S group and labor group within the amnion. (b) *P < 0.05 between C/S group and labor group within the amnion. Ponceau S staining was used as the internal control for Westerns and GAPDH mRNA as the internal control for mRNA.

Western analysis showed that amnion nuclei expressed a higher level of PCAF protein than those from the chorion-decidua. The level was higher in the non-labor group than in the labor group only in the amnion (Figure 5(a)). Real-time RT-PCR revealed that there was no difference in message levels between the amnion and chorion-decidua, but the level was higher in the labor group compared to the non-labor group only in the amnion (Figure 5(b)).

### 3.5. Immunohistochemistry

**Figure 6:**
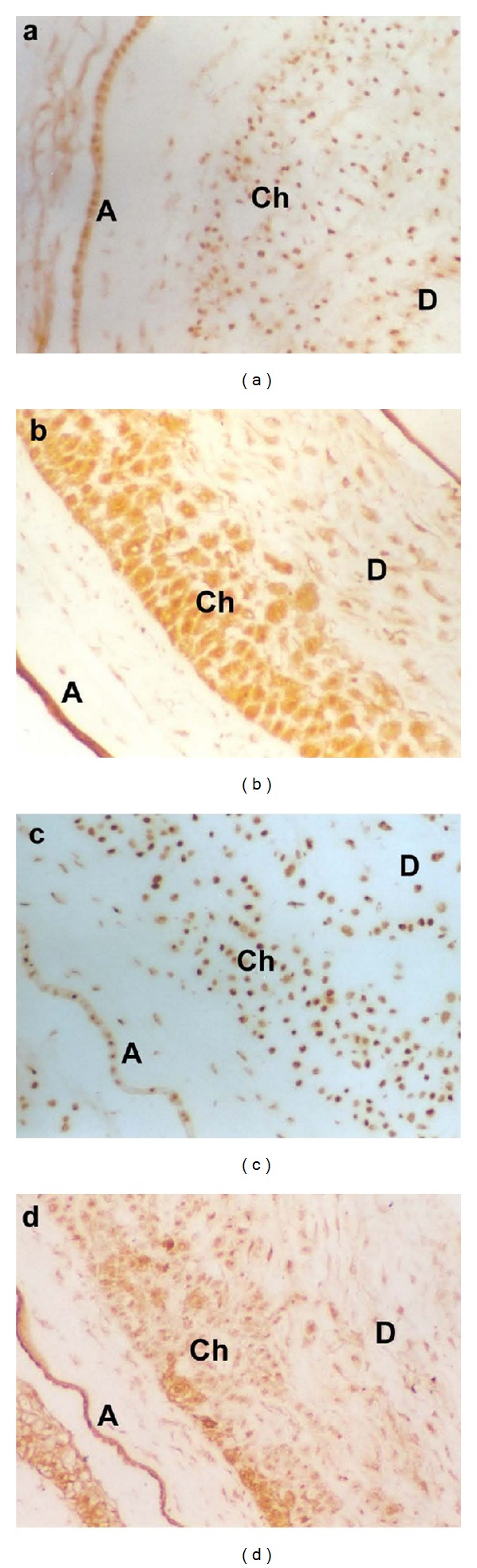
Immunohistochemistry of unseparated fetal membranes stained with antisera specific to SRC-1 (a), SRC-2 (b), p300 (c), and PCAF (d). No counterstain was applied. All pictures were taken at 200× magnification. A: amnion epithelium. Ch: chorion laeve. D: decidua.

Coactivators were localized using antibodies specific to SRC-1, SRC-2, p300, and PCAF. SRC-1 was found to be present primarily in the nuclei of the amnion epithelium, chorion laeve, and decidua (Figure 6(a)). In the case of SRC-2, however, it was present in a large amount both in the nuclei and the cytoplasm in the amnion epithelium and chorion laeve. It was also expressed in the nuclei and cytoplasm in decidual cells, but at a lower level (Figure 6(b)). Similar to SRC-1, p300 was also highly concentrated in the nuclei of all layers (Figure 6(c)), whereas PCAF expression was similar to that of SRC-2 and was localized to both the nuclei and cytoplasm in the amnion epithelium, chorion laeve, and decidua (Figure 6(d)). No difference in staining patterns was found in tissues obtained before and after labor.

## 4. Discussion

We have demonstrated the presence of several nuclear receptor coactivators including SRC-1, SRC-2, p300, and PCAF in the human fetal membranes and decidua. We further showed that these molecules displayed distinct expression patterns and cellular localization within the amnion and chorion-decidua and that these coactivators were temporally regulated around the time of parturition. In contrast to Lappis et al. [25] we did not detect CBP protein in either the amnion or chorion decidua. This was perhaps due to the different methodologies using immunohistochemistry in the previous study [25] and Western analysis in the present study. 

Nuclear receptor coactivators are essential for transcriptional activity of GR and other steroid receptors. We found that two members of the p160 steroid receptor coactivator family, SRC-1 and SRC-2, were expressed in the human fetal membranes. Interestingly, these two coactivators displayed tissue-specific expression; namely, SRC-1 is present predominantly in the amnion, whereas SRC-2 level was higher in the chorion-decidua. Functional studies of knockout mice deficient in SRC-1 and SRC-2 demonstrated distinct phenotypes, indicating that they exhibited different biological functions and that their presence was not functionally redundant [26]. Immunohistochemistry found that SRC-1 was concentrated in the nuclei of amnion epithelium and chorion-decidua. In contrast, SRC-2 was present both in the nuclei and cytoplasm. Whether SRC-1 plays a more important role in steroid responsiveness in these tissues remains to be determined. 

PCAF and p300 belong to another class of nuclear receptor coactivators that possess potent histone acetyl transferase activity. We found that these two molecules were also highly expressed in the human fetal membranes and that, like SRC-1, they were also preferentially expressed in the amnion. Our immunohistochemical analyses found that p300 was almost exclusively nuclear, whereas PCAF was both nuclear and cytoplasmic in these tissues. The nuclear localization of p300 contrasts with a previous study [25] that found p300 localized to the cytoplasm and nucleus. In this latter study the expression levels were lower in membranes distal to the supracervical site compared to the tissue close at the supracervical site but in both sites p300 was localized to the cytoplasm and nucleus.

Our results also demonstrated that after the process of parturition, the protein levels of SRC-1 decreased in the nuclei from the chorion-decidua. Similarly, PCAF protein levels also decreased in the amnion nuclei after the process of parturition. This is to our surprise since we would anticipate that the coactivators increase during labor if they are essential in steroid responsiveness in these tissues. We collected the tissues immediately after the completion of labor and vaginal delivery. We speculate that there may have been a downregulation of these molecules at the time of tissue collection as a result of high levels of cortisol that occur during labor. It has previously been shown that SRC-1 expression can be downregulated by glucocorticoids in vivo and in vitro [27]. It therefore appears crucial in the future to include a group of patients undergoing emergency cesarean section during the process of labor to fully delineate the temporal changes of these coactivators throughout the process of parturition.

In the present study, we found discrepancies between mRNA levels and nuclear protein levels of these coactivators. Message levels of SRC-1, p300, and PCAF were lower in the amnion than in the chorion-decidua, and the reverse was true for SRC-2. This was opposite to nuclear protein expression. These results indicate that posttranscriptional regulation may play a role in determining the final nuclear protein concentrations of these molecules. The posttranscriptional regulatory processes may include mRNA trafficking/transportation, mRNA degradation, translational efficiency, intracellular distribution of the proteins, and protein degradation [27]. For example, PCAF mRNA translation is suppressed by microRNA in liver epithelial cells [28], and p300 protein can be regulated by the proteasome pathway [29]. An alternative explanation could be that the cytoplasmic and nuclear localization seen by us for SRC-2 and PCAF and others for p300 [25] might explain the discrepancies between mRNA levels and nuclear protein. In order to examine this latter possibility, quantitative analysis of both cytoplasmic and nuclear protein will be required.

The expression of nuclear receptor coactivators has been studied in the human myometrium during labor. Condon et al. reported that SRC-2, SRC-3, and CBP messages and proteins in fundal myometrium decreased during active labor in women undergoing emergency cesarean section compared to women having elective cesarean section. They did not find a change in levels of SRC-1 mRNA and protein [20]. These authors suggested that this might represent a mechanism for functional progesterone withdrawal in the human myometrium during parturition. We, however, did not demonstrate similar findings in the human fetal membranes and decidua. The levels of SRC-1 and PCAF proteins decreased in the amnion and chorion-decidua, respectively, after the completion of labor compared to levels prior to the onset of labor. Levels of SRC-2 and p300 proteins did not change while SRC-3 and CBP proteins were not detected by us in these tissues. While the progesterone receptor clearly plays a central role in myometrial activation, its role in the fetal membranes and decidua is less clear. Progesterone receptor isoforms were detected in the decidua but not in the amnion and chorion laeve [30–32]. The absence, or low level, of this receptor in the amnion and chorion laeve at term suggests it is unlikely that the nuclear receptor coactivators present in these tissues are associated with the progesterone receptor. 

These coregulators also interact with other factors believed to be involved in parturition such as NF*κ*B [25], and this has been suggested to play a role in regulating NF*κ*B action at the supracervical site in the fetal membranes in preparation for labor. The differential expression of p300 found in this study [25] suggests that local changes in coregulator expression take place within the membranes and highlights the importance of taking this into account with respect to membrane rupture and cervical ripening. 

## 5. Conclusions

Several of the nuclear receptor coactivators including SRC-1, SRC-2, p300, and PCAF were expressed in the human fetal membranes before and after labor at term. There appeared to be temporal and spatial regulation of these molecules in the amnion, chorion laeve, and decidua. These results were consistent with a complex functional role of these coactivators during parturition in the human fetal membranes.

## Figures and Tables

**Table 1:** Sequences of the primers and TaqMan probes that were used in real-time RT-PCR.

Genes	Primers and probes
SRC-1	Forward: 5′-GGAACCCAGCAGGTGCAA-3′
	Reverse: 5′-CCCGCCTACCAGATTCACTGT-3′
	TaqMan: 5′-AGGTTCAGGTGTTTGCTGACGTCCAGTG-3′

SRC-2	Forward: 5′-AGTGACCTCCGTGCCTACGT-3′
	Reverse: 5′-CTCCCCTCAGAGCAGGATCA-3′
	TaqMan: 5′-CTCCATGGGTCCCGAGCAGG-3′

p300	Forward: 5′-GCTTCTGACAAAACCGTGGAA-3′
	Reverse: 5′-GAAGCACAGGTCAACACCATCA-3′
	TaqMan: 5′-AAGCAAGGTTTGTGGACAGTGGAGAGATG-3′

PCAF	Forward: 5′-GGTTGGCCTACAGAACGTTTTC-3′
	Reverse: 5′-GACGAGCCGTGTGATGTATTCTT-3′
	TaqMan: 5′-ACCAGCTGCCCCGAATGCCA-3′
